# Targeting Early Dementia: Using Lipid Cubic Phase Nanocarriers to Cross the Blood–Brain Barrier

**DOI:** 10.3390/biomimetics3010004

**Published:** 2018-03-07

**Authors:** Joseph S. D’Arrigo

**Affiliations:** Cavitation-Control Technology Inc., Farmington, CT 06032, USA; cavcon@ntplx.net

**Keywords:** Alzheimer’s disease, biomimetic nanocarriers, blood–brain barrier, dementia, drug targeting, lipid cubic phase, nanoemulsion, SR-BI, scavenger receptors

## Abstract

Over the past decades, a frequent co-morbidity of cerebrovascular pathology and Alzheimer’s disease has been observed. Numerous published studies indicate that the preservation of a healthy cerebrovascular endothelium can be an important therapeutic target. By incorporating the appropriate drug(s) into biomimetic (lipid cubic phase) nanocarriers, one obtains a multitasking combination therapeutic, which targets certain cell surface scavenger receptors, mainly class B type I (i.e., SR-BI), and crosses the blood–brain barrier. This targeting allows for various cell types related to Alzheimer’s to be simultaneously searched out for localized drug treatment in vivo.

## 1. Introduction

The fundamental involvement of the cerebrovasculature in the pathogenesis of common dementias, widely reported in the biomedical literature, has recently been reviewed (e.g., [1,2]). Small vessel disease is commonly found in patients who have other brain pathologies, such as plaques and tangles associated with neurodegenerative diseases; small vessel disease also increases the risk of Alzheimer’s disease. Accordingly, vascular cognitive impairment and dementia (VCID) is the second leading cause of dementia, behind Alzheimer’s disease, and is a frequent co-morbidity in Alzheimer’s patients [3,4,5,6,7,8,9]. On a worldwide basis, 47 million people had dementia in 2016; of these dementia patients, 60–80% had Alzheimer’s disease [4,10,11].

## 2. Central Role of Endothelial Dysfunction

It has been reported repeatedly that endothelial modulation and repair is feasible by pharmacological targeting [1,12,13,14,15,16,17,18,19,20,21,22,23,24,25,26] via scavenger receptor class B type I (SR-BI) (cf. [25]). As the detailed review by Mahringer et al. [27] points out, the blood–brain barrier (BBB) is equipped with several endocytic receptors at the luminal surface (i.e., the capillary endothelial membrane), including the SR-BI scavenger receptor. Furthermore, very recently published experimental work (in human-endothelial-cell monolayer cultures as well as in three-dimensional tissue-engineered human vessels) has demonstrated in detail that high-density lipoproteins (HDL), acting via scavenger receptors (specifically SR-BI), block β-amyloid uptake into endothelial cells both in experimental monolayers and probably in the intact human cerebrovascular endothelium [28] (cf. [29,30,31]).

Almer et al. [14] explain in their recent review that the integration of lipoprotein-related or apolipoprotein-targeted nanoparticles, as drug carriers, is an expanding concept in nanomedicine to exploit the intrinsic characteristics of lipoprotein particles as being the natural transporter of lipophilic compounds in human circulation. Discrete lipoprotein assemblies and lipoprotein-based biomimetics offer a versatile nanoparticle platform for constructing drug loaded, reconstituted or artificial lipoprotein particles for specific medical applications. As naturally occurring nanoassemblies, lipoprotein particles are not readily (nor rapidly) cleared by the mononuclear phagocyte system (of the liver and spleen) and remain in circulation for a longer period of time [14]. More recently, Srimanee et al. [12] further explain that receptor-mediated transcytosis (RMT) at the BBB occurs in three steps: (1) receptor-mediated endocytosis at the luminal (capillary endothelial lining/blood) side via ligands (i.e., lipoprotein-related, apolipoprotein-targeted nanoparticles) binding to specific membrane receptors (e.g., SR-BI); (2) transfer of endocytic vesicles through the cytoplasm; (3) and excytosis of the carried (small-molecule or biomolecular) drug at the abluminal (brain/endothelial) side. Currently, several receptors are known to be expressed on the luminal surface of the BBB, which include scavenger receptors (such as SR-BI) [12]. Particularly, SR-BI was found in bovine and porcine brain capillary endothelial cells (BCEC), and also expressed in murine brain. The rodent SR-BI was studied and showed the same structure/behavior as human SR-BI [12,13]. With regard to their own experimental work, Srimanee et al. [12] report that SR-BI are also involved (among several receptor types studied by their group) in the uptake of nanocomplexes into brain endothelial cells, and also mediate the transport of nanocomplexes across their BBB model. Moreover, other published studies have shown that lipophilic compounds bound to HDL (and probably to “HDL-like” nanoparticles as well) have the possibility to be internalized by a “piggy-back”-like mechanism [13]. It was shown that uptake of HDL-associated α-tocopherol by porcine BCEC via SR-BI exceeded the uptake of HDL particles up to 13-fold, suggesting a selective uptake of this compound without the concomitant internalization of the lipoprotein (HDL) particle. Additional work has demonstrated apolipoprotein (apo) A-I expression in porcine brain capillaries [13]. Further research indicated that apoA-I, the major protein component of HDL, was effluxed by porcine BCEC (whereas the aortic endothelium did not efflux any detectable amount of apoA-I). ApoA-I-inducing compounds, such as cholesterol, could upregulate apoA-I in BCEC. These data toegether, suggested that apoA-I is effluxed apparently by the SR-BI receptor in porcine BCEC [13]. Moreover, Fung et al. [32] separately reported that SR-BI mediates the uptake and transcytosis of HDL across brain microvascular endothelial cells (i.e., across the blood–brain barrier). The authors assert that elucidating the mechanisms of HDL transcytosis across the BBB, in particular, may be pathologically significant, as its constituent apoA-I has been demonstrated to confer a protective effect against Alzheimer’s disease. Using a combination of spinning-disc confocal and total internal reflection fluorescence microscopy, these authors examined the internalization and transcytosis of fluorescently labeled HDL by human primary brain microvascular endothelial cell monolayers. Using these approaches, these investigators reported that HDL internalization requires dynamin, but not clathrin heavy chain, and that its internalization and transcytosis are saturable. The authors concluded that these (and other reported) findings indicate that HDL transcytosis across the BBB involves a signaling pathway downstream of SR-BI. These investigators further argue that manipulation of HDL transcytosis across the BBB to increase delivery of plasma apoA-I may, in turn, facilitate increasing the transport of “HDL-like synthetic particles” containing therapeutic drugs across the BBB to treat neurodegenerative disorders such as Alzheimer’s disease [32] (cf. [28,33,34,35,36,37,38,39,40,41,42]).

## 3. Targeted Drug Treatment for Early Dementia

This targeted drug delivery approach, using an apoA-I-based (SR-BI-mediated) therapeutic agent for treating the more common (late-onset) dementias, receives added impetus from continued findings of cerebrovascular pathology [1,43,44,45,46,47,48,49,50,51,52,53] and an apparent endothelium dysfunction [2,33,34,35,36,37,38,39,40,41,49,54,55,56,57,58,59,60] in both Alzheimer’s disease and its major risk factors [1,2,53,54,55,56,57,58,59,60,61,62,63,64,65,66,67,68,69,70,71,72]. By incorporating drug candidates (such as Edaravone, docosahexaenoic acid (DHA), or antibody therapeutics) into the “lipid-coated microbubble/nanoparticle-derived” (LCM/ND) lipid nanoemulsion type (yielding particle sizes mostly <0.1 μm in diameter), known to be a successful drug carrier [73,74], one is likely to obtain a multitasking combination therapeutic capable of targeting cell surface SR-BI. This combination therapeutic would make it possible for various cell types, all potentially implicated in Alzheimer’s disease (cf. [71,72]), to be simultaneously sought out and better reached for localized drug treatment of brain tissue in vivo.

With regard to receptor-mediated membrane transport across the BBB, brain microvascular endothelial cells are believed to control iron uptake and efflux, under the direct guidance of neighboring astrocytes [75,76]. Detailed evidence has been reported recently [75] showing that human brain microvascular endothelial cells, which constitute most of the BBB, receive brain iron status information via paracrine signals from ensheathing astrocytes. Lastly, aging, obesity, and smoking are determinants of brain iron accumulation in human subjects [77] and all have been shown to be associated with the long-term incidence of Alzheimer’s disease [25,50,51,52,54,55,65,78,79,80].

Note that the above-mentioned association of both obesity and diabetes with incidence of Alzheimer’s disease has also renewed the interest in the main facilitative glucose transporter protein in the brain, GLUT-1, and its involvement in and probable contribution to neurodegenerative diseases [81,82,83]. More than two decades ago, it was already recognized that the normal human brain capillary endothelial cells have a high density of GLUT-1, whereas the cerebral microvessels in subjects with Alzheimer’s disease showed a markedly decreased in GLUT-1 density when compared with age-matched controls [84,85]. More recently, Winkler et al. [86] demonstrated that GLUT-1 deficiency in the cerebral endothelium (but not in astrocytes) initiates BBB breakdown in a mouse model of Alzheimer’s disease. These authors observed from their detailed experiments that reduced GLUT-1 expression (at the BBB) worsens Alzheimer’s disease cerebrovascular degeneration, neuropathology, and cognitive function—suggesting that (cerebral endothelial) GLUT-1 may represent a therapeutic target for Alzheimer’s disease vasculo-neuronal dysfunction and degeneration [86]. Furthermore, other investigators [87] (cf. [88]) have recently provided evidence for brain glucose dysregulation as a critical event in Alzheimer’s disease pathogenesis that closely reflects both the severity of the neuropathology and the expression of symptoms in Alzheimer’s disease. Moreover, abnormalities in brain glucose homeostasis may begin several years before the onset of clinical symptoms [87].

In summary, endothelial cells are the main component of the BBB, which is seriously disrupted in various neurological pathologies, including many neurodegenerative disorders [89,90,91]. An early BBB breakdown and/or dysfunction has been documented in Alzheimer’s disease before dementia, neurodegeneration, and/or brain atrophy occur, and investigators have reported that targeting the BBB can influence the course of such neurological disorder [92]. Hence, vascular-targeted therapies become plausible for the prevention and treatment of common dementias [4,36,89,93,94,95]. In respect of vascular tone, vasodilation (mediated by nitric oxide or acetylcholine) is repressed whereas vasoconstriction (mediated by endothelin-1) is enhanced, thus contributing to endothelial dysfunction in Alzheimer’s disease [90,96]. Also, β-amyloid can induce apoptosis and/or necrosis of brain endothelial cells. Presence of β-amyloid, as well as tau protein oligomers, leads to accumulation of inflammatory molecules in microvessels—which further fosters endothelial dysfunction [90,97,98,99]. Other cell types of the neurovascular unit are affected in Alzheimer’s disease as well [90]. For example, deposition and aggregation of β-amyloid within vascular smooth muscle cells leads to inflammation, oxidative stress, impaired vasorelaxation, and disruption of BBB integrity. At the same time, midlife vascular risk factors such as hypertension, cardiovascular disease, diabetes, dyslipidemia, and obesity all increase the relative risk for Alzheimer’s disease [89,100,101,102,103]. These co-morbidities are all characterized by low and/or dysfunctional HDL, which itself is an Alzheimer’s risk factor. Namely (in addition to the widely reported lipid transport), HDL regulates vascular health via modulating vasorelaxation, inflammation, and oxidative stress as well as promoting endothelial cell survival and integrity [36,102,104]. Since SR-BI has already been identified as a major receptor for HDL (with its major constituent apoA-I) as well as for the earlier-described LCM/ND nanoemulsion [1,2], this multitasking lipid nanoemulsion can arguably serve as a targeted, apoA-I-based, (SR-BI-mediated) therapeutic agent for common (late-onset) dementias (cf. [28,33,35,37,38,39,40,41,42]). In this particular targeted delivery approach, the self-assembled HDL-related “lipid nanoemulsion particle” structure itself (after intravenous injection) likely binds to apoA-I in the blood plasma; subsequently, such apoA-I-targeted LCM/ND nanoemulsion particles are recognized by SR-BI receptors on various Alzheimer’s-related cell types [73].

## 4. Lipid-Coated Microbubble/Nanoparticle-Derived Nanoemulsion Type Contains Lipid Cubic Phase Nanocarriers

The self-assembled LCM/ND lipid nanoemulsion comprises nonionic lipids exclusively (cf. [105,106]) throughout its coated microbubbles and/or related nanoparticles (i.e., related lipid polymorphs) supramolecular structures(s). This biobased lipid composition of LCM/ND nanoemulsions (i.e., glycerides and cholesterol compounds) is similar to lipids contained in several types of plasma lipoproteins; accordingly, when these LCM/ND nanoemulsion particles are injected into the bloodstream, they likely acquire (i.e., bind) plasma apolipoprotein(s)—including notably apoA-I [73]. Hence, the molecular composition of the LCM/ND nanoemulsion particles results in both microbubble/nanoparticle stability and marked targeting toward tumors and certain hyperproliferative disease lesions/sites; this very rapid targeting has been demonstrated to occur by an active uptake process, i.e., endocytosis—which likely involves certain “lipoprotein receptor”-mediated endocytic pathways [2].

The collection of powdered solid lipid surfactants used to produce LCM/ND lipid nanoemulsions, which is described with all structural details of the molecular components in the published patents covering this technology [105,106], can be outlined as follows:

- “a member selected from the group consisting of glycerol monoesters of saturated carboxylic acids containing from about 10 to about 18 carbon atoms …;
- a sterol aromatic ester;
- a member selected from the group consisting of sterols …;
- a member selected from the group consisting of sterol esters of aliphatic acids containing from one to about 18 carbon atoms; … and
- a member selected from the group consisting of glycerol, glycerol di-, or triesters of aliphatic acids containing from about 10 to about 18 carbon atoms …”.

“The surfactant mixture of the present invention can be readily prepared by admixing components a through e in a weight ratio a:b:c:d:e of 2–4:0.5–1.5:0.5–1.5:0–1.5:0–1.5, respectively. Preferably … the components of the surfactant mixture of the present invention are combined in a weight ratio a:b:c:d:e of 2–4:1:1:1:1. Since each of the components of the surfactant mixture of the present invention is a dry powder, the resultant admixture is conveniently obtained in a dry powdered form.” In a particularly preferred form (i.e., “Example 1”) of the invention, the “surfactant mixture was prepared in accordance with the present invention by admixing glycerol monolaurate, cholesterol benzoate, cholesterol, cholesterol acetate, and glycerol tripalmitate in a weight ratio of 3:1:1:1:1, respectively, to obtain a dry powdery surfactant mixture” [105,106].

Importantly, monoglyceride is the largest single-lipid fraction (by wt%) of the powdered solid lipid surfactants used to produce the (Filmix^®^) LCM/ND nanoemulsions [73]. As a group, monoglycerides exhibit different phase behaviors when they are exposed to water [107] (cf. [108,109]). The ability to exist in several different phases is an important property of pure lipids and lipid mixtures; it depends upon temperature, hydration, and lipid class [107]. Although monoglycerides typically have poor water solubility, they have free hydroxyl groups which can hydrogen bond with water, surfactants, cosolvents, etc. As polar lipids, monoglycerides typically: (1) are better solvents for drugs; (2) act as cosurfactants which promote mutual solubility between excipients (i.e., inactive ingredients); (3) enhance water uptake; and (4) promote self-dispersibility of lipid formulations [110]. The above properties of monoglycerides place them in a lipid class known as “insoluble swelling amphiphiles”. These lipid molecules form stable monolayers (at the air/water interface), but also swell in water to form liquid crystalline phases [111]. In their detailed review, Kaasgaard and Drummond [112] explain that these lyotropic (i.e., solvent induced) liquid crystalline phases of monoglycerides include the one-dimensional lamellar phase, which has been widely studied and employed as a model system for biomembranes and drug delivery applications. More recently studied are the structurally more complex two- and three-dimensional ordered (lyotropic) liquid crystalline phases, of which inverse hexagonal and cubic phases are two prominent examples. In agreement with numerous other investigators, Kaasgaard and Drummond also state that all these types of liquid crystalline phases are frequently stable in excess water, which facilitates the preparation of nanoparticle dispersions and makes them suitable candidates for the encapsulation and controlled release of drugs ([112]; cf. [113,114,115,116,117,118,119]).

In the “preferred form” of the LCM/ND nanoemulsion formulations (cf. [105,106]), the monoglyceride content employed consists entirely of the saturated variety. Using only saturated monoglyceride in such nanoemulsion formulations carries an additional benefit. Namely, saturated fatty chains (i.e., saturated acyl groups) are advantageous because they are incapable of undergoing peroxidation reactions, which would lessen the acceptable storage life (cf. [120]) of these (“oil-in-water”) nanoemulsions.

The self-assembly of varied and useful dispersed cubic phases (among other liquid crystalline phases) depends heavily on the acyl chain length of the glycerides (primarily monoglycerides) placed in contact with water [73]. As Yaghmur et al. [119] point out, the significant interest in the formulation and the characterization of these complex and varied, self-assembled, liquid crystalline cubic phases is driven by both fundamental and practical considerations: they offer many advantages compared to conventional dispersed systems (such as simple or double emulsions) because of their confined equilibrium nanostructures with high interfacial area, their low viscosity, and their capabilities to solubilize a wide variety of active molecules. Therefore, there is great interest to utilize theses dispersed cubic phases for the administration of drugs, or for the formulation of new delivery systems [119].

The (lyotropic) cubic liquid crystalline phases may be classified into two distinct classes: bicontinuous cubic phases and micellar or discontinuous (e.g., type *Fd3m*) cubic phases. Representative illustrations, including suitable micrographs, of these dispersed cubic phases can be found in [107,112,114,121,122,123,124]. As Abraham et al. [125] explain, two alternate structural representations have been utilized to describe the bicontinuous cubic phases, one in terms of rod-like elements and the other in terms of folded surfaces, that is, infinite periodic minimal surfaces (IPMS) (alternatively, the representations in terms of nodal surfaces have been used to describe the dynamic structure of cubic phases). Three different “inverse bicontinuous” cubic lipid phases have been observed experimentally, having the symmetry *Pn3m*, *Ia3d*, and *Im3m*—corresponding to the following IPMS: the diamond type (D-surface), the gyroid type (G-type), and the primitive type (P-surface), respectively [125]. As reviewed by Garg et al. [107], monoglycerides spontaneously form bicontinuous cubic phases upon the addition of water, are relatively insoluble (allowing the formation of colloidal dispersions of cubic phases), and resistant to changes in temperature. Accordingly, lipid nanoparticles comprising interior liquid crystalline structures of curved lipid membranes (i.e., dispersed cubic phases) have been used to solubilize, encapsulate, and deliver medications to disease areas within the body [107].

Besides certain glyceride-based liquid crystalline systems displaying colloidal stability in excess water, the same important attribute has been documented for cholesterol and cholesterol esters—all of which are present in LCM/ND nanoemulsion formulations [73]. For example, cholesterol and its esters change the packing structure of lipids, and in high concentrations they are known to induce the formation of a liquid crystal phase [120]. In addition, Kuntsche and colleagues [126,127] have prepared lipid nanoparticles in the (mesomorphic or) liquid crystalline phase from cholesterol esters with saturated acyl chains. These investigators were motivated by the knowledge that many cholesterol esters are physiologic lipid compounds which can form liquid crystalline phases (thermotropic mesophases) and, hence, they were interested in their potential for the development of liquid crystalline nanoparticles as a carrier system for lipophilic drugs [127]. In accordance with the above observations and considerations, the substantial concentrations of cholesterol esters and cholesterol in the LCM/ND nanoemulsion formulation likely further contribute to the known long-term stability of this nanoemulsion (liquid crystalline) lipid nanoparticles in excess water, thereby providing a persistent carrier matrix upon exposure to liquids such as blood plasma [73]. 

## 5. Promising Developments Regarding Supplementary Neurotherapy Using Targeted Sonoporation

A completely separate advantage of such LCM/ND (drug delivery) nanoemulsion(s) stems from the characteristic lipid-coated microbubble subpopulation existing in this nanoemulsion type [1,2,73]. Over the past decade, neuroscientists have been exploring the use of ultrasound in combination with preformed (intravenous) microbubbles to temporarily open the BBB (cf. [128,129,130,131,132,133,134,135,136,137,138,139,140,141,142,143,144,145,146,147,148,149]), allowing drugs or the immune system to target brain tumors or Alzheimer’s brain plaque in vivo effectively, repeatedly, and safely [150,151,152,153,154,155,156] in animals up to primates [150,157] and even in humans [157]. It is worth noting that this proposed mechanism of plaque burden reduction, by sonoporation (i.e., “loosening the tight junctions of the cells forming the BBB” via ultrasound irradiation [158,159]), might carry an additional effect. (Microbubble-assisted) sonoporation not only facilitates localized delivery of drugs and/or activated immune cells to target Alzheimer’s brain plaque in vivo [158], but also facilitates (possibly by passive transport) reduction of β-amyloid plaque burden from brain tissue in a mouse model of Alzheimer’s disease [160]. Specifically, this same mechanism might also function to counteract characteristic decreased “brain clearance” of neurotoxic β-amyloid “monomer” [160]—which has been described as a central event in the pathogenesis of Alzheimer’s disease (cf. [1,2,161]).

The actual cellular and biophysical mechanisms of the reversible BBB opening process by sonoporation, when employing focused transcranial ultrasound coupled with injected preformed microbubbles, have been described further in other published studies over the last several years [1,162,163,164,165,166,167,168]. Also, representative illustrations depicting such an opening of the BBB, by postulated loosening of tight junctions (and other mechanisms), can be found in [141,164]. In the foreseeable future, taking full advantage of this ongoing, noninvasive, and targeted use of preformed (LCM/ND nanoemulsion-based) microbubbles to transiently and reversibly increase BBB permeability via sonoporation, while optimizing drug delivery efficiency (through judicious choice of acoustic parameters [152,156]) and minimizing side effects, may assist in advancing transcranial sonoporation to the clinic (cf. [1,167,168,169,170,171,172,173,174,175,176,177,178,179,180,181,182]).

## 6. Lipid-Coated Microbubble/Nanoparticle-Derived Nanoemulsion Particles Function as Biomimetic Cubic Phase Nanotransporters

As alluded to in Section 4, the previously documented similarities in lipid composition among HDL (as well as native and modified low-density lipoproteins (LDL)) and LCM/ND nanoemulsion particles can partially simulate or mimic the known heterogeneity (i.e., subpopulations or subspecies) of HDL particles (for a review, see [73]). Moreover, the above-mentioned scavenger receptor (i.e., either SR-BI (rodent) and/or CLA-1 (human) orthologs [29]) has been shown to be a multifunctional receptor able to bind a broad variety of ligands, including HDL, LDL, oxidized low-density lipoproteins (OxLDL), acetylated low-density lipoproteins (AcLDL), very low-density lipoproteins (VLDL), and chylomicron remnants [183,184,185]. The presence of amphipathic helices is a common feature of “exchangeable apolipoproteins”, which are known to be the primary ligands (including notably apoA-I) for SR-BI [183].

One example of a reconstituted (biomimetic) lipoprotein complex utilizing the SR-BI cell surface receptor in the literature concerns manufactured (lipid) emulsions that were designed to mimic chylomicrons in vivo, and therefore were expected to acquire apolipoproteins upon incubation with serum [186]. The experimental data obtained led investigators to conclude that SR-BI is clearly involved in facilitating chylomicron (remnant) metabolism and might function as an initial recognition site for chylomicron remnants [185]. Note that, in this example, the “reconstituted lipoprotein vehicle” was at first constructed solely of lipids, and apolipoprotein(s) (needed for targeting) were acquired only after incubation with serum. This concept of a pure lipid nanocarrier, which can successfully acquire apolipoprotein(s) upon contact with blood plasma, is similarly described elsewhere in the literature. For example, Williams and Scanu [187] reported that phosphoglyceride liposomes, injected intravenously, pick up endogenous apoA-I; in vitro, phosphoglyceride liposomes incubated with plasma acquire apoA-I at the expense of HDL [73] (cf. [188]). Explanatory illustrations depicting the apoA-I chemical structure, the apoA-I conformation on discoidal and spherical HDL particles, and their relative sizes can be found in [189].

To conclude, self-assembled (colloidal mesophase) lipid nanoemulsions (e.g., [190,191,192,193,194,195]), particularly those predominantly containing dispersed cubic phase lipid nanoparticles (e.g., [196,197,198,199,200]), continue to receive growing attention in pharmaceutical and/or biological fields. The main reason behind much of this attention is the fact that nonlamellar lipid nanostructures, such as cubic liquid crystalline phases, have wide potential as delivery systems for numerous drugs, cosmetics, and food applications (e.g., [201,202,203]). Namely, using various lipids and their mixtures to form self-assembled nonlamellar nanostructures, it has continually been reported possible to successfully obtain stable colloidal dispersions of (liquid crystalline) lipid cubic phases with well-defined particle size and morphology (e.g., [202,203]). In particular, within the range of self-assembled phases in model surfactant-like lipid systems, Yaghmur et al. [204] further emphasized that monoglyceride-based lyotropic liquid crystalline phases are relatively unique owing to their rich polymorphism in water and potential application as drug nanocarriers (cf. [205] and Section 4 above). A recurring example of a largely monoglyceride-based drug delivery agent category (cf. Section 4, Section 5 and Section 6) is the multitasking LCM/ND nanoemulsion formulation. In this particular targeted delivery approach, the self-assembled lipid particle structure itself (upon intravenous injection of the LCM/ND nanoemulsion) is apparently successfully utilized as the active targeting ligand—which is directed via (adsorption of) plasma lipoproteins towards the appropriate receptors on the target cell surface. These dispersed liquid crystalline lipid particles, of the LCM/ND nanoemulsion formulation, are colloidally stable nanocarriers which very likely represent liquid-crystalline inverse-topology nanotransporters (nanocarriers), i.e., dispersed lipid cubic phases (cf. [73]).

## 7. Conclusions

The proposed multitasking combination therapeutic appears likely to display greater efficacy at different stages of Alzheimer’s disease (cf. [72]). Furthermore, the effects on various cell types targeted may be additive, multiplicative, or otherwise synergistic [26]. As a result, this multitasking (drug delivery) therapeutic approach could represent a promising way to treat, delay, or even prevent the disease in the future [1,2]. In particular, LCM/ND (lipid) nanoemulsion particles have a composition (consisting of various glycerides, cholesterol, and cholesterol esters) similar to lipids contained in several plasma lipoproteins (i.e., resembles the lipid content of a “generic” lipoprotein [184,190]). Accordingly, when this specific nanoemulsion type is injected intravenously, its colloidally stable lipid particles apparently acquire apoA-I from the plasma and, subsequently, can be recognized by and bind to certain lipoprotein receptors (predominantly SR-BI) on various Alzheimer’s-related cell types.
